# Cilia Dynamics in Primary Ciliary Dyskinesia: A Biophysical Characterization of the *RSPH4A* Founder Variant

**DOI:** 10.3390/cells15070607

**Published:** 2026-03-29

**Authors:** Gabriel Rosario-Ortiz, José Muñiz-Hernández, Natalia M. Ortiz-Pérez, Marcos J. Ramos-Benitez, Ricardo A. Mosquera, Wilfredo De Jesús-Rojas

**Affiliations:** 1 Department of Pediatrics and Basic Science, Ponce Health Sciences University, Ponce, PR 00716, USA; grosario24@stu.psm.edu (G.R.-O.); nortiz22@stu.psm.edu (N.M.O.-P.); mjramos@psm.edu (M.J.R.-B.); 2 San Juan Bautista School of Medicine, Caguas, PR 00725, USA; josemh@sanjuanbautista.edu; 3 Department of Pediatrics, McGovern Medical School at UTHealth Houston, Houston, TX 77030, USA; ricardo.a.mosquera@uth.tmc.edu

**Keywords:** primary ciliary dyskinesia, high-speed video-microscopy analysis, ciliary beat pattern, cilia physics, biophysics

## Abstract

Primary ciliary dyskinesia (PCD) is a rare ciliopathy resulting in chronic oto-sino-pulmonary disease. PCD diagnosis can be achieved by a combination of different diagnostic and adjuvant tools, including high-speed video-microscopy analysis (HSVA). A founder variant has been described in Puerto Rico as the most common cause of PCD in the island. Background/Objectives: In HSVA, objective parameters such as ciliary beat frequency (CBF) and subjective parameters such as ciliary beat pattern (CBP) shed light on the biophysical properties of cilia. However, the subjective nature of CBP creates a gap in knowledge; characteristics such as the length, angle, and bending index of cilia are poorly described. Our goal is to quantify cilia dynamics of the *RSPH4A* (c.921+3_921+6delAAGT (intronic)) founder variant in Puerto Rico through biophysical properties of cilia. This approach enhances longitudinal patient care by understanding treatment progress through biophysical ciliary function. Methods: We analyzed images from HSVA of six patients with PCD homozygous for the founder variant and six healthy controls (HC) (*n* = 12). Results: We found that ciliary length (PCD = 7.62 ± 0.95 μm, HC = 8.12 ± 1.36 μm, *p* = 0.204 ns), orientation vector (PCD = 7.20 ± 0.93 μm, HC = 7.25 ± 1.01 μm, *p* = 0.883 ns), straight angle (PCD = 1.67 ± 0.27 rad, HC = 1.76 ± 0.29 rad, *p* = 0.380 ns), and area (PCD = 2.35 ± 0.52 μm^2^, HC = 2.10 ± 0.53 μm^2^, *p* = 0.264 ns) did not have statistically significant differences between PCD and HC. In contrast, bending index (PCD = 1.06 ± 0.04, HC = 1.12 ± 0.09, *p* = 0.01), bent angle (PCD = 1.11 ± 0.30 rad, HC = 0.67 ± 0.21 rad, *p* < 0.0001), net angle (PCD = 0.56 ± 0.26 rad, HC = 1.09 ± 0.35 rad, *p* < 0.0001), amplitude (PCD = 5.77 ± 1.25 μm, HC = 7.99 ± 1.65 μm, *p* < 0.0001), and amplitude per second (PCD = 48.83 ± 13.23 A(s), HC = 91.66 ± 27.96 A(s), *p* < 0.0001) showed significant differences between both groups. Conclusions: Reduced angular excursion and amplitude in PCD demonstrate that the beating pattern of the *RSPH4A* founder variant is dysfunctional as compared with healthy controls. Our study provides an objective framework to understand the biophysical properties of the *RSPH4A* founder variant.

## 1. Introduction

Primary ciliary dyskinesia (PCD) is a rare genetic disorder that affects ciliary motility, compromises mucociliary clearance (MCC), and predisposes to chronic upper and lower respiratory infections, chronic rhinosinusitis, and bronchiectasis [1]. Diagnosing PCD is complex, requiring clinical assessment plus specialized diagnostic and adjuvant tests to confirm or exclude it [2,3]. The American Thoracic Society and European Respiratory Society guidance endorses a multi-modal diagnostic algorithm that integrates characteristic clinical features with nasal nitric oxide (nNO), high-speed video-microscopy analysis (HSVA) of ciliary beat frequency (CBF) and ciliary beat pattern (CBP), immunofluorescence, transmission electron microscopy (TEM), and comprehensive genetic testing [1,2,4].

Among these modalities, HSVA provides both quantitative and qualitative assessments that underlie genetic and ultrastructural abnormalities. While CBF alone has limited specificity, CBP can discriminate normal from dyskinetic or immotile motion and often reveals subtle, genotype-specific signatures, including in cases with normal ultrastructure [5,6,7]. Accordingly, CBP analysis has emerged as a critical bridge between genotype and phenotype in PCD. *RSPH4A* encodes a radial spoke head protein that is required for coordinated axonemal regulation and effective ciliary beating. Pathogenic variants can therefore cause dysfunction of the radial spoke and central apparatus, resulting in an abnormal ciliary beat phenotype. Importantly, abnormalities in radial spoke and central apparatus have also been described in non-*RSPH4A* genotypes [7]. For example, *RSPH1* variants have been reported to show an abnormal rotational beat pattern despite relatively preserved CBF, illustrating that impaired MCC may result from beat pattern inefficiency rather than frequency reduction alone [8]. Recent reviews similarly describe rotational motion as a recurring feature of some radial spoke/central complex defects, including *RSPH1* and *RSPH9* [9]. In contrast, other PCD genotypes tend to exhibit different HSVA signatures, such as complete immobility, often seen with combined dynein arm defects. Stiff or low amplitude beating has been associated with inner dynein arm and axonemal ruler–related defects, such as *CCDC39* and *CCDC40*. Hyperkinetic but ineffective beating has been reported in some cases with *DNAH11* variants [7].

In Puerto Rico, a unique cohort has been identified with the intronic *RSPH4A* genetic variant (c.921+3_921+6delAAGT), being responsible for approximately 70% of PCD cases [10,11,12]. This genetic variant is linked to recurrent respiratory infections without laterality defects. It exhibits a distinctive ciliary motion profile characterized by reduced CBF and a unique rotational pattern [10,12,13]. Population screening indicates a high allele frequency and considerable underdiagnosis, with an estimated 1624 affected individuals on the island [14].

The clinical consequences and characteristics of CBF related to this founder variant have been extensively documented [12]. However, the detailed biophysical dynamics of the *RSPH4A* variant remain unexplored. Therefore, our study aimed to objectively analyze the cilia dynamics linked to this PCD variant in Puerto Rico by examining the biophysical properties of cilia using ex vivo nasal biopsies. By gaining insights into these parameters, we aim to establish a threshold that distinguishes healthy individuals from patients carrying the *RSPH4A* Puerto Rican founder variant.

## 2. Materials and Methods

### 2.1. Video Collection

A total of 144 high-speed video-microscopy recordings were obtained and analyzed, corresponding to 12 videos per patient. For each patient, recordings included 10 lateral-view videos and 2 top-view videos. The videos were obtained from the CILIA4PR Research Team’s cilia databank at the Ponce Research Institute in Puerto Rico.

From the 10 lateral-view recordings obtained per patient, the three highest-quality videos were selected for analysis based on the clear visualization of a single identifiable cilium. Selection criteria required that the cilium be distinctly observable in both phases of the power stroke, enabling the extraction of two representative frames (one per phase) and the generation of a superimposed image.

Quantitative measurements were performed on these selected videos, and the mean value for each variable was calculated across the three videos per patient. In total, 36 lateral-view videos (3 per patient) were included in the final analysis. For top-view recordings, both videos obtained per patient were included for analysis. These videos were used to measure the area of an individual cilium. The mean value of the measurements from the two videos was calculated for each patient. A total of 24 top-view videos (2 per patient) were analyzed. In summary, although 144 videos were initially evaluated, a subset of 60 videos met the predefined quality criteria and were included in the final quantitative analysis, comprising 36 lateral-view videos and 24 top-view videos.

### 2.2. Video Processing

All videos were recorded at 500 fps, DIC with the AOS PROMON U750 mono-chrome high-speed camera (AOS Technologies AG, Baden-Daettwil, Switzerland) at 40× (NA 0.60) magnification using the Nikon Eclipse Ti2 inverted microscope (Nikon Corporation, Tokyo, Japan). Nasal biopsy samples were centrifuged and resuspended in Medium 199. A 100 μL drop was placed into a microscopy slide to begin the HSVA. To ensure adequate visualization of the ciliary epithelium, we analyzed 10 lateral views and 2 top views per patient. Two specific frames from each video that offered an unobstructed view of individual cilia were taken. The first frame was taken at the point where the cilium initiated its power stroke, referred to as the straight angle. The second frame was taken when the cilia were closest to the epithelial surface, defined as the bent angle. These frames were then superimposed to create a composite image, which allowed for a precise determination of the net angle, or the angle difference of the first two angle parameters. Lastly, a top-view image of the ciliary epithelium was taken to determine the area that an individual cilium occupies.

### 2.3. Variables Studied

Multiple ciliary parameters were evaluated from the images: The orientation vector was defined as the vector length starting from the base of the cilia at the epithelium up to its end point. Length was defined as the complete length of the cilium from the base to its end point, taking its bend into consideration. Straight angle and bent angle represented the angles at the start and end of the ciliary power stroke. Net angle was the angular difference between the straight and bent angles. Amplitude, like the net angle, was determined using the superimposed composite image. This parameter referred to the distance between the end points of the straight angle and the bent angle. The area of cilia was understood as the space occupied by a single cilium seen from a top-view perspective. Other parameters, such as the bending index and amplitude per second, were determined by making calculations based on the values obtained from FIJI (ImageJ v2.16.0/1.54p), the software analysis tool used to measure all physical parameters [15]. The bending index was determined by dividing the length by the orientation vector. Amplitude per second was calculated by multiplying the amplitude by the CBF. The CBF was determined by doing a manual count with each video following previously published guidelines [16]. Table 1 is a summary of our measurements with their respective mathematical units.

### 2.4. Software Analysis

FIJI, a widely used image-processing software, was employed to quantify the parameters extracted from the HSVA images [15]. This software enabled precise measurement of the ciliary dimensions, angles, and areas using its built-in analytical tools. The superimposed images were manually analyzed to determine the straight and bent angles, net angle, and amplitude. Additionally, FIJI’s vector measurement tools were used to calculate orientation vectors, length, and bending index. The top-view images of the cilia were also processed through FIJI to measure the area. CBF was calculated by manually counting the number of complete ciliary beats in a video sequence, following previously published guidelines.

### 2.5. Statistical Analysis

All continuous data are presented as mean ± standard deviation (SD). Figures to represent our findings were created using BioRender (latest available web-based version), and all statistical analyses were performed using the statistical software package GraphPad Prism version 10.1.1 for MacOS, developed by GraphPad Software, San Diego, CA, USA [www.graphpad.com (accessed on 10 July 2024)]. Significance between the HC and PCD groups was determined using unpaired *t*-tests. A *p*-value of <0.05 was considered statistically significant, with values represented as ns (non-significant) for *p* > 0.05, (*p* < 0.05), (*p* < 0.01), (*p* < 0.001), or (*p* < 0.0001).

## 3. Results

### 3.1. Structural Morphology

**Figure 1:**
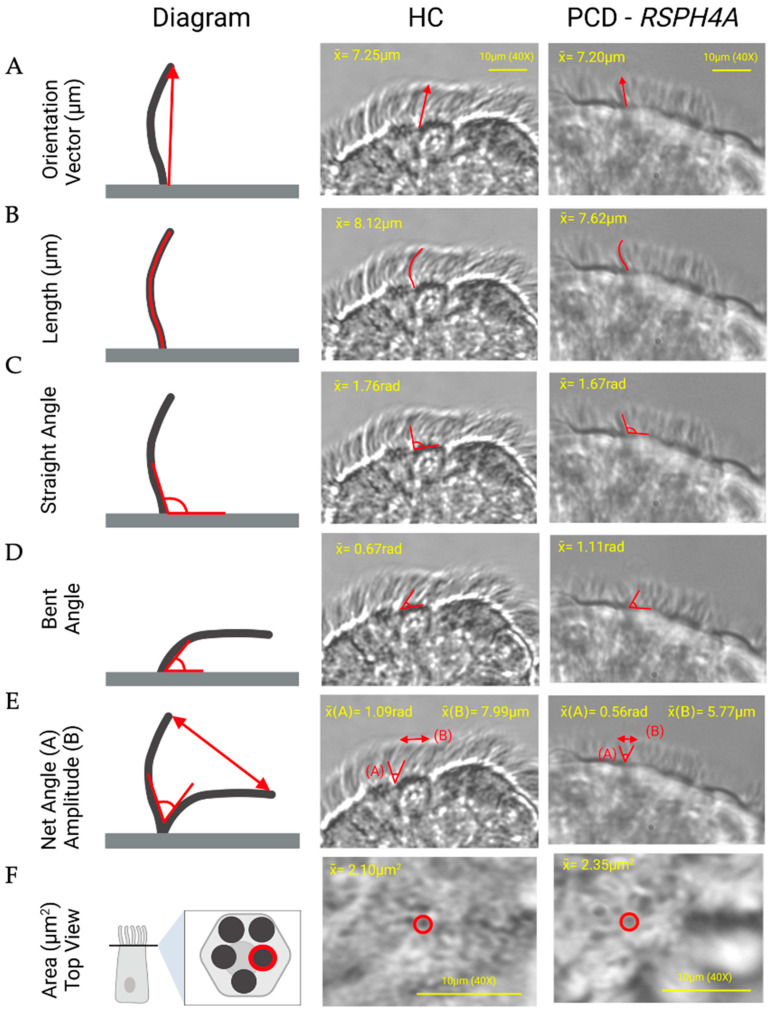
Comparison of ciliary parameters between healthy controls (HC) (*n* = 6) and primary ciliary dyskinesia (PCD) patients (*n* = 6) with the *RSPH4A* founder variant. Key biophysical properties: (**A**) orientation vector, (**B**) length, (**C**) straight angle, (**D**) bent angle, (**E**) bending index, and (**F**) area. Schematic diagrams (left panel) define each parameter, while representative frames from HC and PCD–*RSPH4A* samples (right panels) demonstrate measurement application. HC cilia exhibit coordinated, directional motion with preserved amplitude, whereas PCD–*RSPH4A* cilia show reduced displacement, abnormal angulation, and disorganized beating patterns. Still frames are shown for reference.

**Figure 2:**
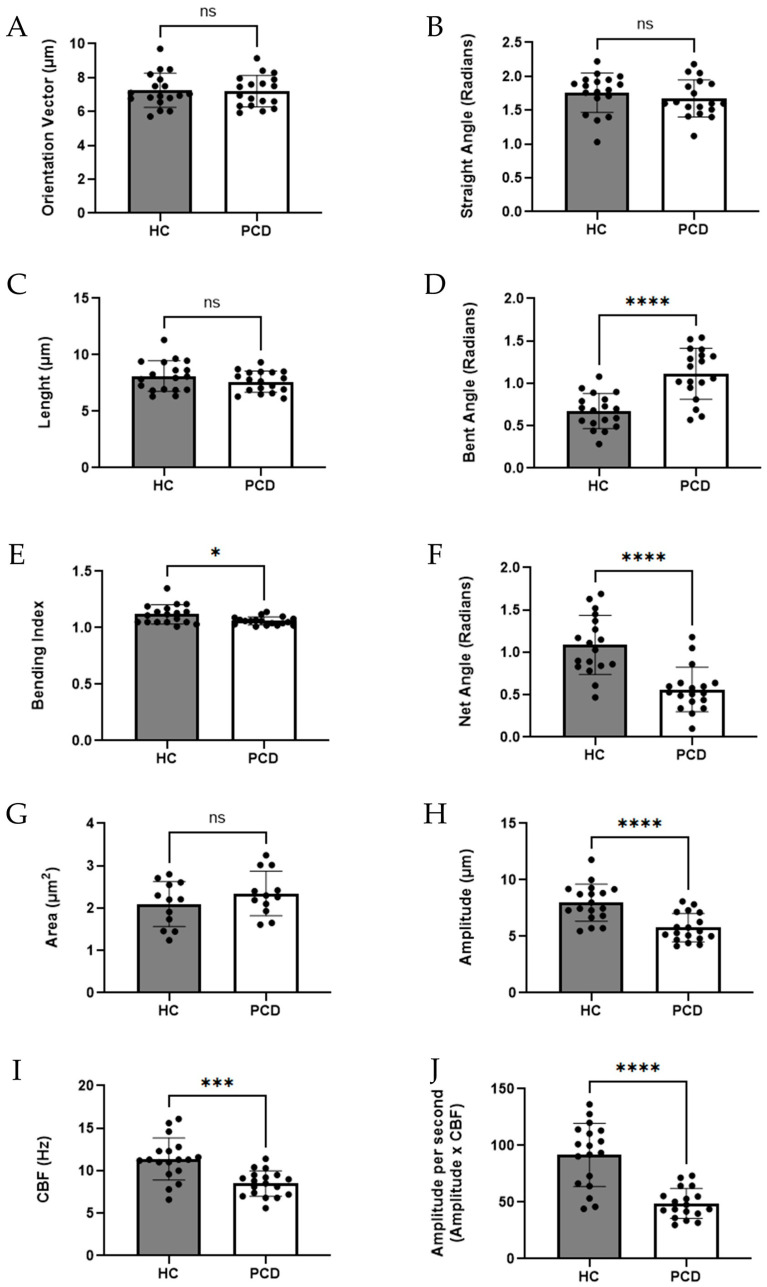
Quantitative comparison of ciliary biophysical dynamics between HC, (*n* = 6) and patients with PCD, (*n* = 6) carrying the *RSPH4A* founder variant. Individual data points are shown with bars representing mean ± SD. (**A**) Orientation vector, (**B**) straight angle, (**C**) length, (**D**) bent angle, (**E**) bending index, (**F**) net angle, (**G**) ciliary area (top-view), (**H**) amplitude, (**I**) ciliary beat frequency (CBF), and (**J**) amplitude per second (amplitude × CBF). Structural parameters (**A**–**C**,**G**) showed no significant differences between groups. In contrast, dynamic parameters (**D**–**F**,**H**–**J**) demonstrated significant alterations in PCD, including increased bent angle, reduced net angle, decreased amplitude, lower amplitude per second, and reduced CBF, reflecting impaired ciliary motility. Significance levels are indicated as follows: ns, not significant (*p* > 0.05); * *p* < 0.05; *** *p* < 0.001; **** *p* < 0.0001.

Figure 1 and Figure 2 present a comprehensive overview of these measurements with corresponding statistical values. Ciliary dimensions were constant when comparing patients with PCD-*RSPH4A* founder variant and HC. The orientation vector, which describes the straight-line distance from the base to the tip of the cilia (Figure 1A), showed no statistically significant difference between patients with PCD: 7.20 ± 0.93 μm and HC: 7.25 ± 1.01 μm, *p* = 0.883 (Figure 2A). The ciliary length was similar, PCD: 7.62 ± 0.95 μm versus HC: 8.12 ± 1.36 μm, *p* = 0.204, which did not significantly differ between groups (Figure 2B).

### 3.2. Ciliary Dynamics and Parameters

Analysis of the ciliary motion properties revealed several significant differences in our cohort. The bending index, which quantifies how much the cilia bend during motion, was reduced, PCD: 1.06 ± 0.04 versus HC: 1.12 ± 0.09, *p* = 0.01, proving a significant decrease in axonemal flexibility in the PCD group (Figure 1C). When analyzing the ciliary area from a top-view perspective, no significant differences were detected with PCD: 2.35 ± 0.52 μm^2^ versus HC: 2.10 ± 0.53 μm^2^, *p* = 0.264 (Figure 2F). Other dynamic properties revealed profound changes in ciliary function, like the bent angle PCD: 1.11 ± 0.30 rad versus HC: 0.67 ± 0.21 rad, *p* < 0.0001, and the net angle PCD: 0.56 ± 0.26 rad versus HC: 1.09 rad ± 0.35, *p* < 0.0001 (Figure 2C,D).

**Figure 3:**
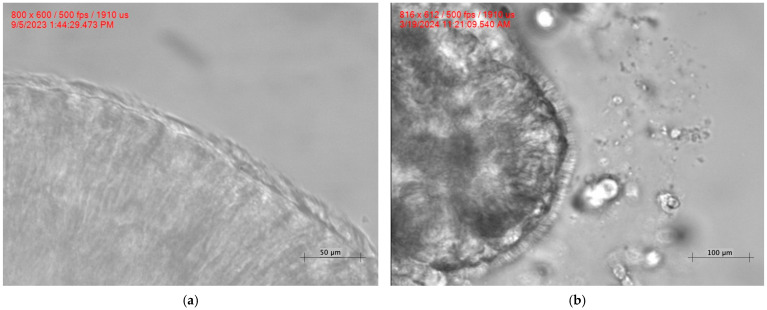
(**a**) Normal beating cilia (CBF: 12 Hz) with a clean apical surface due to effective MCC. (**b**) Impaired MCC in PCD-*RSPH4A*. Dyskinetic cilia (CBF: 8.1 Hz) are unable to clear secretions from the apical surface of the respiratory epithelium. A video demonstrating the biophysical dynamics of the *RSPH4A* (c.921+3_921+6del (intronic)) founder variant (Video S2) compared with healthy control cilia (Video S1) is available in the Supplemental Material.

The amplitude, which measures the distance between the straight and bent positions of the cilia, was markedly lower in patients with PCD: 5.77 ± 1.25 μm versus. HC: 7.99 ± 1.65 μm, *p* < 0.0001, further indicating impaired motility (Figure 1D). Additionally, the amplitude per second A/s PCD: 48.83 ± 13.23 A(s) versus HC: 91.66 ± 27.96 A(s), *p* < 0.0001, showed a reduction (Figure 1E). Finally, CBF itself was reduced in patients with PCD: 8.48 ± 1.50 Hz; HC: 11.38 ± 2.48 Hz; *p* = 0.0002 (Figure 2F). To visually exhibit the implications of our findings in cilia dynamics, Figure 3 provides a clear view of MCC between a normal HC cilia epithelium and PCD-*RSPH4A* ciliary epithelium.

## 4. Discussion

This study demonstrates that Puerto Rican patients with PCD carrying the *RSPH4A* (c.921+3_921+6delAAGT (intronic)) founder variant exhibit profound, quantifiable abnormalities in ciliary beat biomechanics despite preserved gross structural dimensions. Specifically, ciliary length, orientation vector, and top-view ciliary surface area were comparable to healthy controls, supporting that the primary defect in this genotype is not driven by major changes in cilia size or alignment. In contrast, multiple dynamic parameters were significantly abnormal, including bent angle, net angle, amplitude, amplitude per second, bending index, and ciliary beat frequency (CBF). Together, these results provide objective evidence of dysfunctional ciliary biomechanics in this founder variant and offer a mechanistic basis for impaired MCC.

The combination of an exaggerated bent angle and reduced net angle suggests that although cilia undergo substantial curvature during motion, the effective forward component of the power stroke is restricted. This pattern is consistent with impaired coordination of axonemal activity, a feature commonly associated with central apparatus functional disruption and radial spoke abnormalities [9]. In the context of *RSPH4A*, these findings align with the reported rotational CBP, in which beating may appear active but fails to generate productive, directional mucus transport [12].

The reductions in amplitude and amplitude per second indicate diminished mechanical output of the ciliary beat over time, consistent with reduced force generation and impaired beat pattern effectiveness. Physiologically, these abnormalities are expected to translate into weak forward propulsion of mucus and reduced MCC efficiency [7]. This functional consequence is supported by Figure 3, which demonstrates apical mucus retention, consistent with impaired clearance.

A lower bending index suggests altered ciliary flexibility and/or abnormal axonemal stiffness, indicating that the cilium may not achieve the deformation patterns required for an efficient and coordinated beat. Given the role of the radial spoke head complex in organizing dynein-driven microtubule sliding and stabilizing beat pattern propagation, dysfunction at this level can plausibly shift the mechanical properties of the axoneme toward less efficient curvature to transport coupling, producing a beat that is visually apparent but mechanically unproductive [17,18].

CBF was also reduced in the founder-variant group. However, CBF alone is unlikely to fully explain the phenotype, as clinically significant transport failure can occur even with relatively preserved frequency when beat pattern mechanics are abnormal [8]. In this cohort, the concurrent abnormalities in angles, amplitude-derived metrics, and bending index indicate a broader disruption in beat mechanics. Therefore, dynamic beat pattern parameters provide critical explanatory value beyond CBF, helping to clarify why mucociliary transport is impaired.

Our findings align with HSVA studies showing that radial spoke head defects (including *RSPH1*, *RSPH9*, and *RSPH4A*) frequently result in abnormal CBP, often described as rotational [18]. Prior reports have largely characterized these patterns qualitatively, whereas our study provides quantitative biophysical measurements of key dynamic outputs (angles, amplitude, bending index, and time-normalized mechanical parameters) [19]. This objective data helps bridge ultrastructural observations often associated with radial spoke/central apparatus perturbation commonly linked to 9+0 configurations or central apparatus defects with measurable mechanical consequences. Collectively, our results reinforce the principle of genotype-specific CBP and extend it to genotype-specific ciliary biomechanics. The combination of reduced amplitude and abnormal angular dynamics is expected to markedly reduce mucus transport efficiency. The functional correlation shown in Figure 3 supports this relationship by demonstrating mucus retention consistent with compromised MCC in patients with the *RSPH4A* founder variant.

Persistent impairment in MCC promotes mucus accumulation, recurrent infection, and chronic inflammation, contributing to progressive airway injury and the development of bronchiectasis. These quantified biomechanical abnormalities therefore provide a plausible mechanism linking the *RSPH4A* founder variant to long-term pulmonary morbidity observed in this population. Clinically, this is consistent with the characteristic PCD phenotype associated with radial spoke defects, including recurrent respiratory infections and a tendency toward absence of laterality defects [9,12].

Quantitative biomechanical parameters may improve HSVA interpretation, especially in genotypes where ultrastructure can be normal or near normal, making diagnosis challenging when relying solely on qualitative descriptors [1,6]. A standardized panel of biophysical metrics (e.g., net angle, amplitude, bending index, and amplitude/sec) could enhance reproducibility across laboratories. Because these outputs are computationally tractable, they also support automation, including AI-assisted diagnostic tools that improve consistency and make ciliary motion classification more scalable.

The Puerto Rican *RSPH4A* founder variant provides a unique opportunity to study ciliary mechanics in a relatively homogeneous genotype, reducing biologic variability from mixed-variant cohorts and strengthening genotype-specific inference [9,13]. This population, therefore, serves as a valuable model for understanding how radial spoke head dysfunction reshapes the biophysical outputs of the ciliary beat and results in clinically relevant impairment in MCC.

Key strengths include (i) the first objective quantification of dynamic ciliary mechanics associated with *RSPH4A*, (ii) use of high-resolution HSVA paired with FIJI analysis to derive reproducible biophysical parameters, (iii) evaluation of a homogeneous founder variant population that reduces confounding by mixed genotypes, and (iv) integration of structural, dynamic, and functional perspectives into a single framework that links genotype to mechanical output and physiologic consequence.

## 5. Limitations

Several limitations should be acknowledged. The sample size is small, reflecting constraints inherent to rare disease research and limiting generalizability. Imaging constraints, including reliance on standard magnification and recording conditions compared with ultra-high-speed platforms, may reduce sensitivity to subtle beat pattern features. HSVA images of PCD samples exhibit diminished tissue image quality with mild transparency in Figure 1. Differences in visual quality might be related to continuous tissue damage caused by PCD symptoms. Video analyses were not conducted in a blinded manner with respect to group assignment, which may introduce observer bias. Manual frame selection introduces operator dependence and potential selection bias. In addition, variability in biopsy sampling depth and epithelial health may influence measured dynamics. Finally, the retrospective design and lack of longitudinal follow-up limit our ability to directly associate these biomechanical parameters with disease progression over time.

## 6. Future Directions

Future work should validate these findings in larger cohorts, including *RSPH4A* genotypes outside Puerto Rico, to determine generalizability and variant-specific effects. The development of automated or semi-automated pipelines to quantify angles, amplitude, and bending index would improve scalability and reduce operator dependence. Integrating mechanical outputs with nNO, immunofluorescence, and multi-omics (e.g., proteomics) may enable deeper genotype-to-endotype classification. Finally, linking ciliary biomechanics with clinical outcomes (e.g., bronchiectasis severity, exacerbation frequency, FEV1 decline, and proteomics) will clarify prognostic value and may support use of these metrics as objective end points to evaluate response to therapies such as hypertonic saline, airway clearance, chronic macrolide regimens (e.g., azithromycin), and emerging anti-inflammatory strategies (e.g., brensocatib, where applicable).

## 7. Conclusions

This study provides the first quantitative characterization of the biophysical ciliary dynamics associated with the Puerto Rican *RSPH4A* (c.921+3_921+6delAAGT (intronic)) founder variant. While the overall ciliary dimensions, including length, orientation vector, and surface area, were preserved, patients demonstrated profound abnormalities in dynamic parameters, including reduced net angle, amplitude, bending index, amplitude per second, and ciliary beat frequency. These findings indicate that the mechanical efficiency of the ciliary power stroke is markedly impaired despite preserved structural dimensions, reflecting a dysfunctional ciliary beat pattern unique to this radial spoke defect. By integrating objective, reproducible measurements derived from HSVA with FIJI-based image analysis, our work moves beyond subjective pattern interpretation and establishes a quantitative framework to distinguish healthy individuals from those with PCD due to the *RSPH4A* founder variant. This approach strengthens diagnostic precision and provides a foundation for future studies evaluating longitudinal change, genotype-specific mechanisms, and the impact of therapeutic interventions on ciliary biomechanics.

## Figures and Tables

**Table 1:** This table summarizes the key biophysical parameters of cilia analyzed in the study, providing a detailed explanation of each characteristic along with its corresponding unit of measurement. These variables, including orientation vector, length, angles during motion, and beat frequency, are critical to understanding the functional dynamics of cilia motion in both HC and patients with PCD, particularly in relation to the *RSPH4A* founder variant. The definitions serve to clarify the method of quantifying ciliary structure and function.

Characteristics	Meaning	Units
Orientation Vector	Vector from the base of cilia to the end point	Micrometers (μm)
Length	Complete length of cilia taking into consideration bending	Micrometers (μm)
Bending Index	Length divided by orientation vector	Dimensionless ratio
Straight Angle	Angle while initiating stroke motion	Radians (rad)
Bent Angle	Angle while finishing stroke motion	Radians (rad)
Net Angle	Difference between straight and bent angle	Radians (rad)
Amplitude	Length between end point in straight angle and end point in bent angle	Micrometers (μm)
Amplitude Per Second	Amplitude multiplied by the CBF	Amplitude per second (A/s)
Area From Top-View	Circular area of cilia observed from above	Area (μm^2^)
CBF	Ciliary beat frequency	Hertz (Hz)

## Data Availability

The original contributions presented in this study are included in the article/Supplementary Material. Further inquiries can be directed to the corresponding author.

## Supplementary Materials

The following supporting information can be downloaded at: https://www.mdpi.com/article/10.3390/cells15070607/s1. Video S1: Normal beating cilia (CBF: 12 Hz) with a clean apical surface due to effective mucociliary clearance. Video S2: Impaired mucociliary clearance in PCD-*RSPH4A*. Dyskinetic cilia (CBF: 8.1 Hz) are unable to clear secretions from the apical surface of the respiratory epithelium.
